# The Dose-Dependent Organ-Specific Effects of a Dipeptidyl Peptidase-4 Inhibitor on Cardiovascular Complications in a Model of Type 2 Diabetes

**DOI:** 10.1371/journal.pone.0150745

**Published:** 2016-03-09

**Authors:** Ju-Young Moon, Jong Shin Woo, Jung-Woo Seo, Arah Lee, Dong Jin Kim, Yang-Gyun Kim, Se-Yeun Kim, Kyung Hye Lee, Sung-Jig Lim, Xian Wu Cheng, Sang-Ho Lee, Weon Kim

**Affiliations:** 1 Division of Nephrology, Department of Internal Medicine, Kyung Hee University, College of Medicine, Seoul, Korea; 2 Division of Cardiology, Department of Internal Medicine, Kyung Hee University, College of Medicine, Seoul, Korea; 3 Department of Pathology, Kyung Hee University, College of Medicine, Seoul, Korea; University of Edinburgh, UNITED KINGDOM

## Abstract

**Objective:**

Although dipeptidyl peptidase-4 (DPP-4) inhibitors have been suggested to have a non-glucoregulatory protective effect in various tissues, the effects of long-term inhibition of DPP-4 on the micro- and macro-vascular complications of type 2 diabetes remain uncertain. The aim of the present study was to investigate the organ-specific protective effects of DPP-4 inhibitor in rodent model of type 2 diabetes.

**Methods:**

Eight-week-old diabetic and obese db/db mice and controls (db/m mice) received vehicle or one of two doses of gemigliptin (0.04 and 0.4%) daily for 12 weeks. Urine albumin excretion and echocardiography measured at 20 weeks of age. Heart and kidney tissue were subjected to molecular analysis and immunohistochemical evaluation.

**Results:**

Gemigliptin effectively suppressed plasma DPP-4 activation in db/db mice in a dose-dependent manner. The HbA1c level was normalized in the 0.4% gemigliptin, but not in the 0.04% gemigliptin group. Gemigliptin showed a dose-dependent protective effect on podocytes, anti-apoptotic and anti-oxidant effects in the diabetic kidney. However, the dose-dependent effect of gemigliptin on diabetic cardiomyopathy was ambivalent. The lower dose significantly attenuated left ventricular (LV) dysfunction, apoptosis, and cardiac fibrosis, but the higher dose could not protect the LV dysfunction and cardiac fibrosis.

**Conclusion:**

Gemigliptin exerted non-glucoregulatory protective effects on both diabetic nephropathy and cardiomyopathy. However, high-level inhibition of DPP-4 was associated with an organ-specific effect on cardiovascular complications in type 2 diabetes.

## Introduction

Type 2 diabetes is a worldwide problem, and the prevalence and incidence are increasing strikingly even in developing countries. All current efforts are devoted to the control of hyperglycemia to prevent the development of micro- and macro-vascular complications. However, glycemic management in type 2 diabetic patients has become increasingly complex, and new concerns about the effects of intensive glycemic control on the development of cardiovascular complications have arisen. Ultimately, cardiovascular complications are heterogeneous both pathogenetically and in individual patients. Comprehensive cardiovascular risk reduction must be a major focus of therapy, and an increasing array of relevant pharmacological agents is becoming available.

Recently, drugs affecting the incretin system have been introduced and evaluated. Oral dipeptidyl peptidase 4 (DPP-4) inhibitors enhance the circulating concentrations of the active hormone incretin, glucagon-like peptide-1 (GLP-1), and glucose-dependent insulinotropic peptide (GIP). The major effects of these peptides appear to be regulation of insulin and glucagon secretion from the pancreas. In addition, DPP-4 inhibitors exert pleiotropic effects. The GLP-1 receptor, a G-protein-coupled receptor, is expressed in pancreatic islet cells and in the kidney, lung, brain, gastrointestinal tract, and heart [1]. Importantly, recent studies have suggested that DPP-4 inhibitors exert organ-protective effects in models of type 1 or 2 diabetic kidney injury and in models of renal and cardiac ischemia-reperfusion. However, two recent clinical trials of saxagliptin and alogliptin showed that neither drug reduced the risk of cardiovascular events and may even have increased the risk of heart failure in patients with type 2 diabetes mellitus [2,3]. To explore these issues, we investigated the effects of a DPP-4 inhibitor, gemigliptin, on diabetic nephropathy and cardiomyopathy. We showed that the DPP-4 inhibitor exhibited dose-dependent organ-specific effects on the cardiovascular complications of a type 2 diabetes model.

## Methods

### Animal Model and Experimental Design

Six-week-old male non-diabetic db/m and diabetic db/db mice were purchased from the Jackson Laboratory (Sacramento, CA, USA). All mice received a diet of rodent pellets (348 kcal/100 g) containing 5.5% crude fat and tap water *ad libitum*. Gemigliptin (LG life Science, Daejeon, Korea) was incorporated into chow as the concentration of 0.04% and 0.4%. This was calculated to reach an oral dose of 30–300 mg/kg body weight/day, respectively, at a chow consumption of 0.1g/g. At 8 weeks of age, the mice were divided into four groups of eight mice each: the non-diabetic control (db/m), the diabetic group (db/db), a diabetic group treated with 0.04% gemigliptin-treated group (db/db + 0.04% GG), and 0.4% gemigliptin-treated group (db/db + 0.4% GG) from 8 to 20 weeks of age. All animal experiments were approved by the Committee for the Care and Use of Laboratory Animals in Kyung Hee University, Seoul, Korea.

### Measurement of Laboratory Parameters

Food and water intake, urine volume, body weight, and fasting plasma glucose and HbA1c levels were measured monthly. HbA1c was estimated via immunoassay (the DCA 2000 system; Bayer Diagnostics, Elkhart, IN, USA). To measure urinary albumin excretion, individual mice were placed in a metabolic cage, and urine was collected over 24 hours. The urinary microalbumin concentration was determined using a competitive enzyme-linked immunoabsorbent assay (ALPCO, Salem, NH, USA) and normalized to the urinary creatinine level. Urinary 8-OHdG levels were measured by ELISA (Enzo Life Sciences, Lausen, Switzerland).

Plasma DPP4 enzymatic activity was assayed using glycyl-prolyl-7-amino-4-methylcoumarin (AMC) as a fluorogenic substrate. The concentration of free AMC was measured FlexStation II^384^ (Molecular devices, USA) using 360 nm excitation/460 nm emission respectively.

### Echocardiography

Transthoracic echocardiography was performed at the end of the study (20 weeks), prior to sacrificing the animals, to obtain two-dimensional M-mode images using a 12-MHz linear probe (Vivid Q; GE Medical Systems, Milwaukee, WI, USA). Mice were anesthetized with ketamine, the chests shaved carefully, and ultrasound gel placed on each thorax to allow the endo- and epi-cardial borders of the heart to be visualized clearly. M-mode images of the left ventricle (LV) were obtained from parasternal short-axis (at the level of the papillary muscles) and parasternal long-axis views. LV cavity size was measured during at least three beats in each projection and averaged. The M-mode images yielded systolic and diastolic wall thicknesses (anterior and posterior) and LV end-systolic and end-diastolic diameters (LVESD and LVEDD, respectively). LV fractional shortening was calculated as below equation.

$$\frac{\left(LVEDD\;-LVESD\right)}{LVESD}*100$$

The ejection fraction was calculated using the Teichholz formula.

### Light Microscopy

For light microscopy, kidney tissue was embedded in paraffin, cut into 3-μm-thick sections, and stained with the periodic acid-Schiff (PAS) reagent. PAS-stained areas of at least 30 glomeruli from each section were quantified using the KS-400 version 4.0 image analysis system (Carl Zeiss Vision, Munich, Germany). The extent of glomerular mesangial matrix expansion for each glomerulus was evaluated semiquantitatively using a scoring system ranging from 0–4: grade 0, no lesion; grade 1, <25%; grade 2, 25–50%; grade 3, 50–75%; and grade 4, >75%. At least 50 glomeruli per section were analyzed in a blinded manner under ×200 magnification.

Cardiac tissue was embedded in paraffin prior to histological evaluation of the cardiomyocyte sectional area and fibrosis. Tissue sections 5 μm in thickness were deparaffinized, rehydrated, and stained with hematoxylin and eosin (H&E) to determine cardiomyocyte areas. To visualize cardiac fibrosis, sections were stained with Sirius red. The proportions of interstitial and perivascular fibrosis were calculated as the ratios of Sirius red-positive to total tissue areas, determined via color-based thresholding [4]. Images from all stained cardiac tissue sections were analyzed using Image J software.

### Immunohistochemistry and TUNEL Staining

Immunohistochemical procedures were performed on 3-μm-thick sections using the Bond Polymer Intense Detection system (Vision BioSystems, Melbourne, Australia), according to the manufacturer’s instructions, with minor modifications. In brief, 3-μm-thick sections of formalin-fixed paraffin-embedded tissues were deparaffinized in Bond Dewax solution (Vision BioSystems), and antigen retrieval was achieved by incubation in the Bond ER solution (Vision BioSystems) for 30 min at 100°C. Endogenous peroxidase was quenched by incubation with hydrogen peroxide for 5 min. Sections were incubated for 15 min or overnight at ambient temperature with primary polyclonal antibodies against nephrin (1:100, ENZO Life Sciences, NY, USA), WT-1 (1:100, Santa Cruz Biochemicals, Santa Cruz, CA, USA), CD31 (1:100, Abcam, MA, USA), and α-SMA (1:100, Abcam, MA, USA) followed by a biotin-free polymeric horseradish peroxidase-linked antibody conjugate system in a Bond-maX automatic slide stainer (Vision BioSystems). Nuclei were counterstained with hematoxylin or nuclear fast red. The extent of glomerular nephrin expression was evaluated by dividing the positively stained area by the total glomerular tuft area. The numbers of WT1-positive nuclei per glomerulus were counted. At least 20 glomeruli per sample were examined at ×400 magnification. The number of capillaries and arterioles were counted and expressed as density per millimeter of tissue.

Cells that had entered advanced stages of apoptosis were detected with a TUNEL assay, performed with a commercial fluorometric TUNEL system kit (Promega, Madison, WI), according to the manufacturer’s instructions. For tubular and glomerular apoptotic scores, TUNEL-positive cells were screened, counted and analyzed using a fluorescence microscope in at least 10 random fields per kidney.

### Western Blot Analysis

Kidney and heart tissues were washed in phosphate-buffered saline (PBS) and lysed in ice-cold lysis buffer (10 mM Tris·HCl, 150 mM NaCl, 1% Triton X-100, 5 mM EDTA [pH 8.0]), containing a protease inhibitor cocktail (Roche Diagnostics, Mannheim, Germany). Lysates were centrifuged at 4°C for 10 min at 10,000 g and the supernatants recovered. Equal amounts of total cellular protein were subjected to SDS-PAGE in 12% acrylamide gel and transferred to PVDF membranes (Millipore) via electroblotting; the membranes were blocked with 5% fat-free milk in Tris-buffered saline with 0.5% Tween 20 (TBS-T). The membranes were incubated overnight at 4°C with primary antibodies against β-actin, iNOS, Nox2, Nox4, p22-phox, p47-phox, p67-phox (all 1:1,000, Santa Cruz Biochemicals), phosphorylated Akt, total Akt, phosphorylated FoxO3a, total FoxO3a, PI3K, phosphorylated GSK3β, total GSK3β, Bax, Bcl-2, cleaved caspase-3, and p53 (all 1:1000, Cell Signaling Technology, MA, USA) dissolved in TBS-T with 5% bovine serum albumin (BSA). The blots were next washed and incubated with secondary antibody in blocking solution (goat anti-rabbit antibody conjugated with horseradish peroxidase [HRP] and goat anti-mouse antibody conjugated with HRP; both 1:10,000; Santa Cruz Biochemicals) for 1 h at room temperature. Signals were detected using a pico-enhanced peroxidase detection (EPD) Western blot kit (Mbiotech, Seoul, Korea), and bands were visualized with the aid of the G:Box Chemi-XL kit (Syngene, Cambridge, UK). β-actin served as internal control.

### Statistical Analysis

All values are expressed as means ± SEMs. Data were analyzed using the Kruskal-Wallis nonparametric test for multiple comparisons. Any significant differences detected were confirmed using the Wilcoxon rank sum and Mann-Whitney tests (to compare the mean differences); P-values <0.05 were considered to be statistically significant.

## Results

### The Effects of Gemigliptin on Plasma HbA1c, DPP-4 Levels and GLP-1 Activity

**Fig 1:**
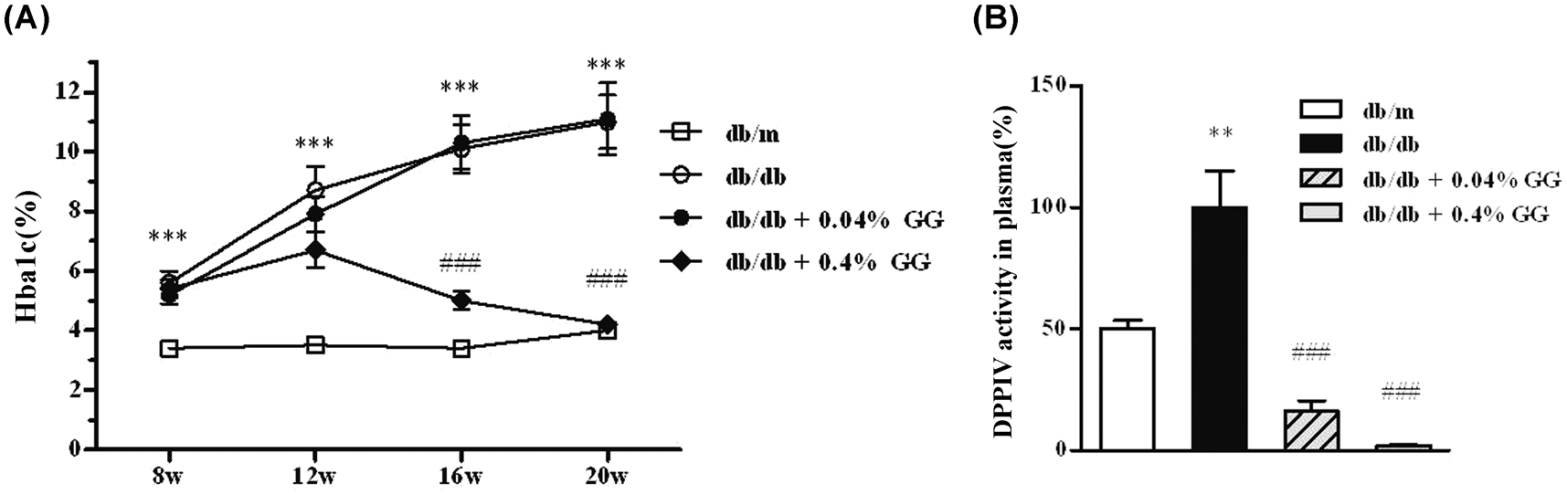
The effect of gemigliptin on HbA1c levels and plasma DPPIV activities in db/db mice. HbA1c levels in the plasma (A). Plasma DPPIV activities in db/m, db/db, and db/db mice treated with 0.04% (w/w) or 0.4% (w/w) gemigliptin (B). All results are expressed as means ± SEMs. **p < 0.01, ***p < 0.001 vs. db/m; ###p < 0.001 vs. db/db.

We compared various biochemical parameters among four experimental groups of mice (Table 1). At 12 weeks post-treatment, the 0.4% gemigliptin-treated group exhibited significantly lower fasting glucose and HbA1c levels. However, we found no difference in either fasting glucose or HbA1c levels between db/db mice and those of the 0.04% gemigliptin group (Table 1, Fig 1). Only the 0.4% gemigliptin-treated group exhibited a significantly lower kidney-to-body weight ratio and serum triglyceride level. Gemigliptin effectively decreased plasma DPP-4 activities (db/db; 100.0 ± 15.1% vs. 0.04% GG; 16.1 ± 4.3%; 0.4% GG; 2.2 ± 0.1%).

**Table 1:** Physical and biochemical characteristics of study groups.

	db/m (n = 8)	db/db (n = 8)	db/db + GG 0.04% (n = 8)	db/db + GG 0.4% (n = 8)
Body weight (g)	29.9±3.8	47.8±11*	57.2±6.4*	52.5±2.7*
Daily food intake (g/day)	5.2 ±0.2	7.5 ±0.5*	7.2 ±0.4*	8.4 ±0.2*
Daily water intake (mL/day)	4.9 ±0.2	13.3 ±0.6*	8.5 ±0.5*^#^	2.1 ±0.2*^#^
Fasting glucose (mg/dL)	84±43	392±81*	326±156*	99±18^#^
AST (IU/L)	119±34	256±276	127±47	110±12
ALT (IU/L)	54±17	175±147	163±124	108±126
Triglyceride (mg/dL)	94±33	228±161	181±61	36±2
BUN (mg/dL)	28.1±5.9	21.0±8.4	22.5±1.6	22.1±10.9
Serum Creatinine (mg/dL)	0.40±0.05	0.45±0.08	0.5±0.01	0.4±0.02
Kidney/BW (%)	0.92±0.04	1.20±0.18*	0.95±0.19	0.7 ±0.2^#^
Liver/BW (%)	4.5±0.2	5.5 ±0.5*	6.5 ±0.3*	5.3 ±0.1*
Fat/BW (%)	2.9±0.5	4.7±0.3*	4.7±0.8*	5.0±0.2*

Abbreviation: GG, gemigliptin

The results are expressed as the mean ± S.E.M.

*Significantly different with respect to the **db/m** mice;

^#^significantly different with respect to **db/db** mice

*^#^*P* < 0.05.

### Gemigliptin Regulates Diabetic Cardiac Myopathy

Echocardiography revealed dilated LVs and reduced cardiac performance in animals with type 2 diabetes. The 0.04% gemigliptin-treated group exhibited a significant reduction in the extent of LV dilatation, as indicated by reduced LV diastolic and systolic dimensions and volume. Both the LV ejection fraction and fractional shortening were improved upon administration of 0.04% gemigliptin. However, 0.4% gemigliptin did not significantly improve either LV dilatation or dysfunction (Table 2).

**Table 2:** Gemigliptin ameliorates left ventricular dysfunction in diabetes.

	db/m (n = 8)	db/db (n = 8)	db/db + GG 0.04% (n = 8)	db/db + GG 0.4% (n = 8)
IVS thickness (mm)	0.75 ± 0.10	0.74 ± 0.08	0.67 ± 0.02	0.65 ± 0.07
PW thickness (mm)	0.81 ± 0.08	0.79 ± 0.14	0.67 ± 0.05	0.71 ± 0.08
LVEDD (mm)	3.95 ± 0.31*	4.62 ± 0.27	4.13 ± 0.30*	5.04 ± 0.25
LVESD (mm)	2.57 ± 0.33*	3.32 ± 0.36	2.82 ± 0.23*	3.84 ± 0.40
LVEDV (mL)	0.16 ± 0.04*	0.25 ± 0.04	0.18 ± 0.04*	0.32 ± 0.05
LVESV (mL)	0.04 ± 0.02*	0.10 ± 0.02	0.06 ± 0.01*	0.15 ± 0.05
EF (%)	70.5 ± 5.5*	61.1 ± 6.9	66.8 ± 3.7	53.8 ± 7.8

Abbreviation: IVS indicates interventricular septum; PW, posterior wall; LVEDD, left ventricular end-diastolic dimension; LVESD, left ventricular end-systolic dimension; LVEDV, left ventricular end-diastolic volume; LVESV, left ventricular end-systolic volume, EF; ejection fraction.

The results are expressed as the mean ± S.E.M.

*p<0.05 (vs db/db)

**Fig 2:**
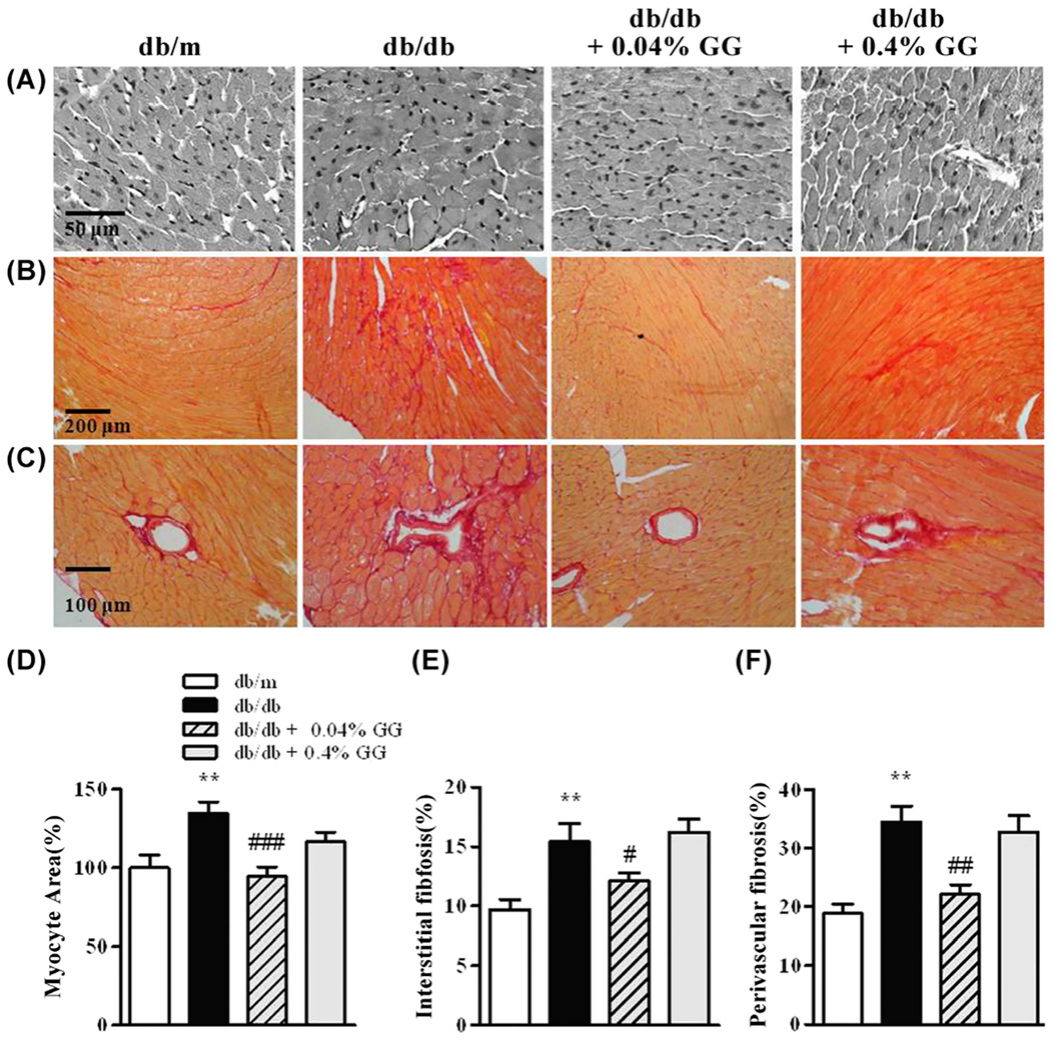
Gemigliptin (0.04%) attenuates myocyte hypertrophy and fibrosis in the diabetic heart. Representative images of H&E-stained cardiac tissues (A), and Sirius red-stained interstitial (B) and perivascular areas (C). Quantification of cardiomyocyte numbers in cross-sectional tissue areas (D). The proportions of interstitial (E) and perivascular (F) tissues. Data were analyzed by one-way ANOVA using Newman-Keuls post-hoc testing, and the results are expressed as means ± SEMs. **p < 0.01 vs. db/m; #p < 0.05, ##p < 0.01, ###p < 0.001 vs. db/db.

**Fig 3:**
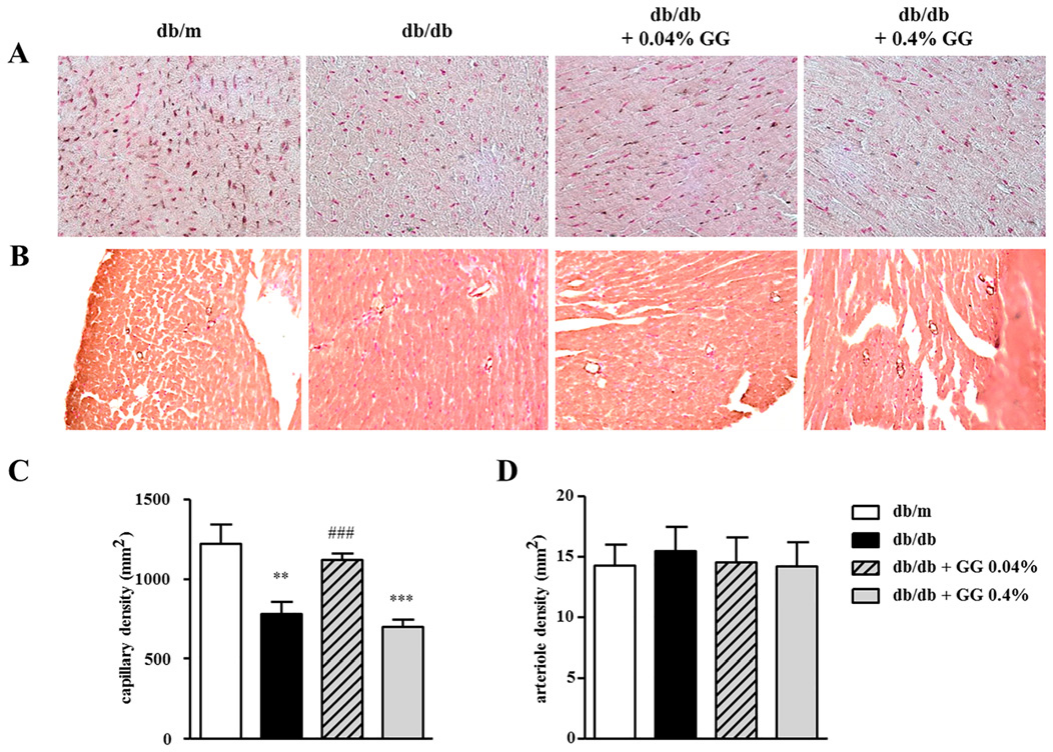
The effect of gemigliptin on myocardial capillary and arteriole density in db/db mice. Representative immunohistochemical stain showing myocardial capillary (CD31) and arteriole (α-SMA) density (A and B). Quantitative analysis of the capillary and arteriole density in myocardium was shown (C and D). Data were analyzed by one-way ANOVA using Newman-Keuls post-hoc testing, and the results are expressed as means ± SEMs. **p < 0.01 vs. db/m; ***p < 0.001 vs. db/m; ###p < 0.001 vs. db/db.

The hearts of diabetic mice exhibited hypertrophy, including increased areas of cardiac myocytes, and interstitial and perivascular fibrosis (Fig 2A, 2B and 2C). The cardiomyocyte cross- sectional area was decreased in the 0.04% gemigliptin-treated db/db mice compared with untreated controls (Fig 2D). Furthermore, interstitial (Fig 2E) and perivascular (Fig 2F) fibrosis were also attenuated in the 0.04% gemigliptin-treated db/db heart. In contrast, the histological extent of cardiac hypertrophy was not influenced by 0.4% gemigliptin. To assess the influence on diabetic microvascular complication of gemigliptin, the heart tissue was stained with anti-CD31 (Fig 3A) or α-SMA (Fig 3B) antibodies. Cardiac capillary density was determined by counting CD31-positive structures and the arteriole density was defined as α-SMA-positive vessels. Cardiac capillary density was reduced under diabetic conditions, which was suppressed by 0.04% gemigliptin but not by 0.4% gemigliptin (Fig 3C), while the arteriole density did not altered by both concentration of gemigliptin (Fig 3D). Therefore, gemigliptin ameliorated diabetic cardiac myopathy at a concentration of 0.04% but not 0.4%.

### Gemigliptin Attenuates Apoptosis in the Diabetic Heart

**Fig 4:**
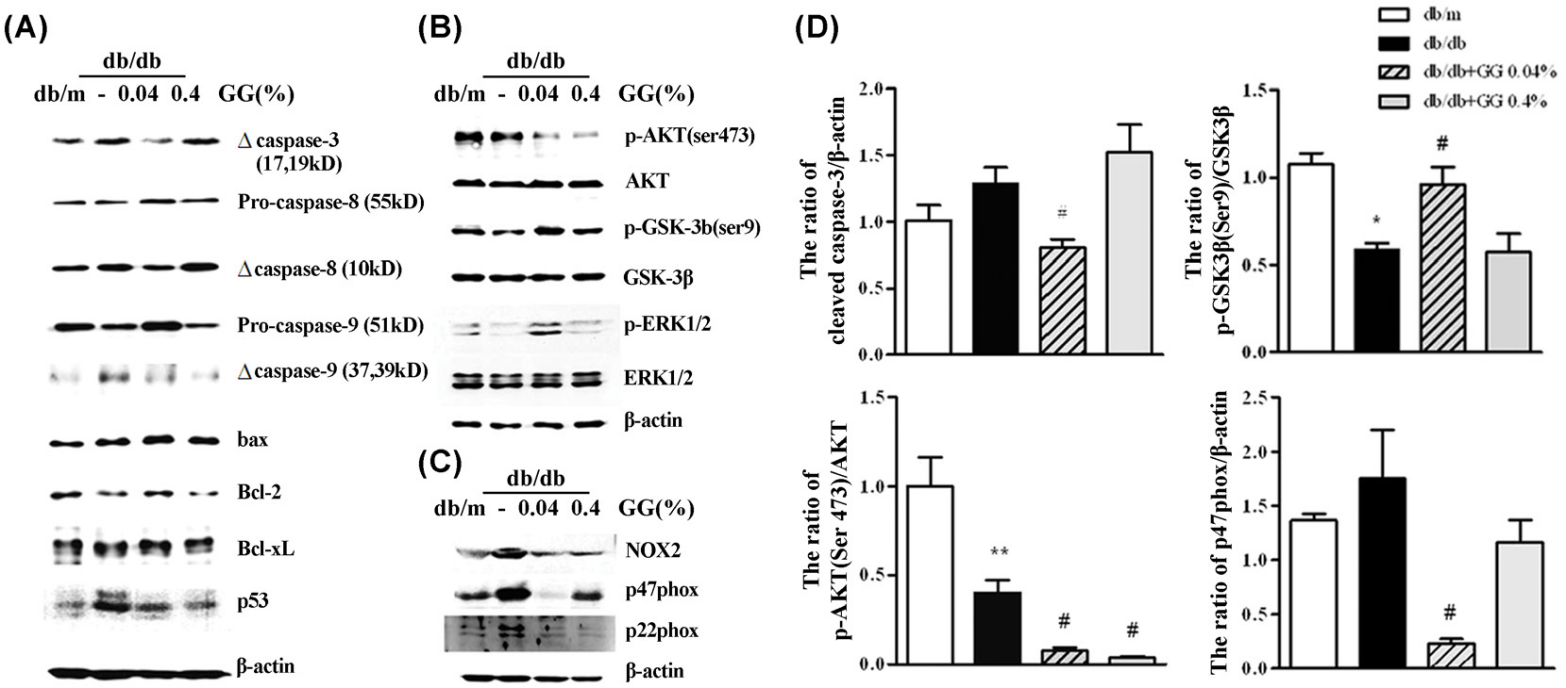
Gemigliptin (0.04%) attenuates apoptosis in the diabetic heart. Representative Western blot analysis of apoptosis-associated protein levels (A). The levels of phosphorylated AKT and GSK3β (B) and of NADPH oxidase subunits (C). A bar graph (means ± SEMs) showing the changes in three or four animals per group (D). *p < 0.05, **p < 0.01 vs. db/m; #p < 0.05 vs. db/db.

To evaluate the effect of gemigliptin on cardiac myocyte apoptosis in diabetic heart, we examined apoptosis-related protein expression in diabetic heart. The enhanced cleavage of caspase-3 was decreased in the 0.04% gemigliptin treated diabetic heart, and caspase-8 and -9 cleavage also prevented by 0.04% gemigliptin. Bcl-2 level was increased by only 0.04% gemigliptin treatement and the Bax/Bcl2 ratio (data not shown) was decreased when 0.04% gemigliptin was subjected (Fig 4A).

Although Akt-GSK3β plays an important role in prevention of cardiac apoptosis, Akt phosphorylation decreased by gemigliptin in a dose-dependent manner. However, GSK3β serine 9 phosphorylation was stimulated only in 0.04% gemigliptin treated diabetic heart (Fig 4B). To examine the effect of gemigliptin on diabetic ROS modulating in heart, we evaluated the expression level of components of NAD(P)H oxidase complex. Increased expression of NOX2, p47phox and p22phox were diminished by 0.04% gemigliptin (Fig 4C). However, GSK3β serine 9 phosphorylation was not stimulated and p47phox was not attenuated by 0.4% gemigliptin.

### Gemigliptin Reduces Albuminuria Independently of Glycemic Control

**Fig 5:**
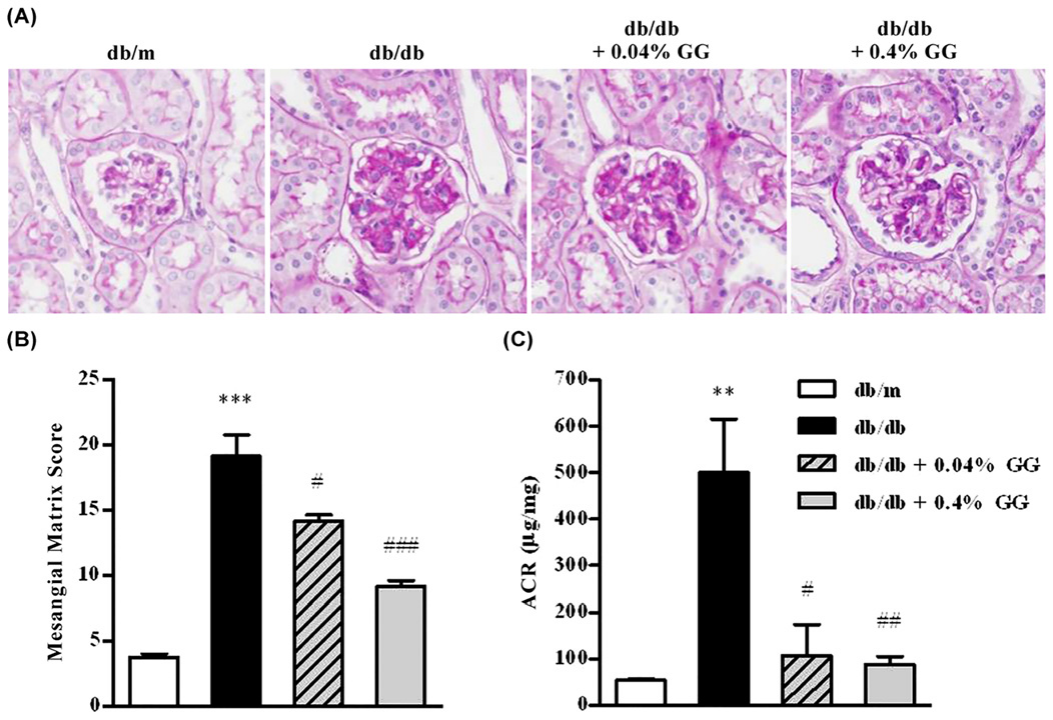
The effects of gemigliptin on mesangial matrix expansion and urinary albumin/creatinine ratios in db/db mice. Mesangial expansion was explored by PAS staining of the kidneys of a) db/m, b) db/db, c) db/db + 0.04% (w/w) gemigliptin-, and d) db/db + 0.04% (w/w) gemigliptin-treated mice (A). Quantitative analysis of glomerular matrix scores (B). Urinary albumin/creatinine ratios (C). The results are expressed as means ± SEMs. **p < 0.01, ***p < 0.001 vs. db/m; #p < 0.05, ##p < 0.01, ###p < 0.001 vs. db/db. Original magnification ×400.

The urinary albumin/creatinine ratio was significantly decreased in both the 0.04% and 0.4% gemigliptin-treated groups compared with db/db controls (Fig 5C). As shown in Fig 5A and 5B, gemigliptin attenuated mesangial expansion in a dose-dependent manner. These findings suggest that 0.04% gemigliptin exerted a non-glucoregulatory renoprotective effect on diabetic nephropathy. However, 0.4% gemigliptin prevented the development of nephropathy along or in company with the normalized glycemic control.

### Both of 0.04 and 0.4% Gemigliptin Prevent Podocyte Injury and Apoptosis in the Diabetic Kidney

**Fig 6:**
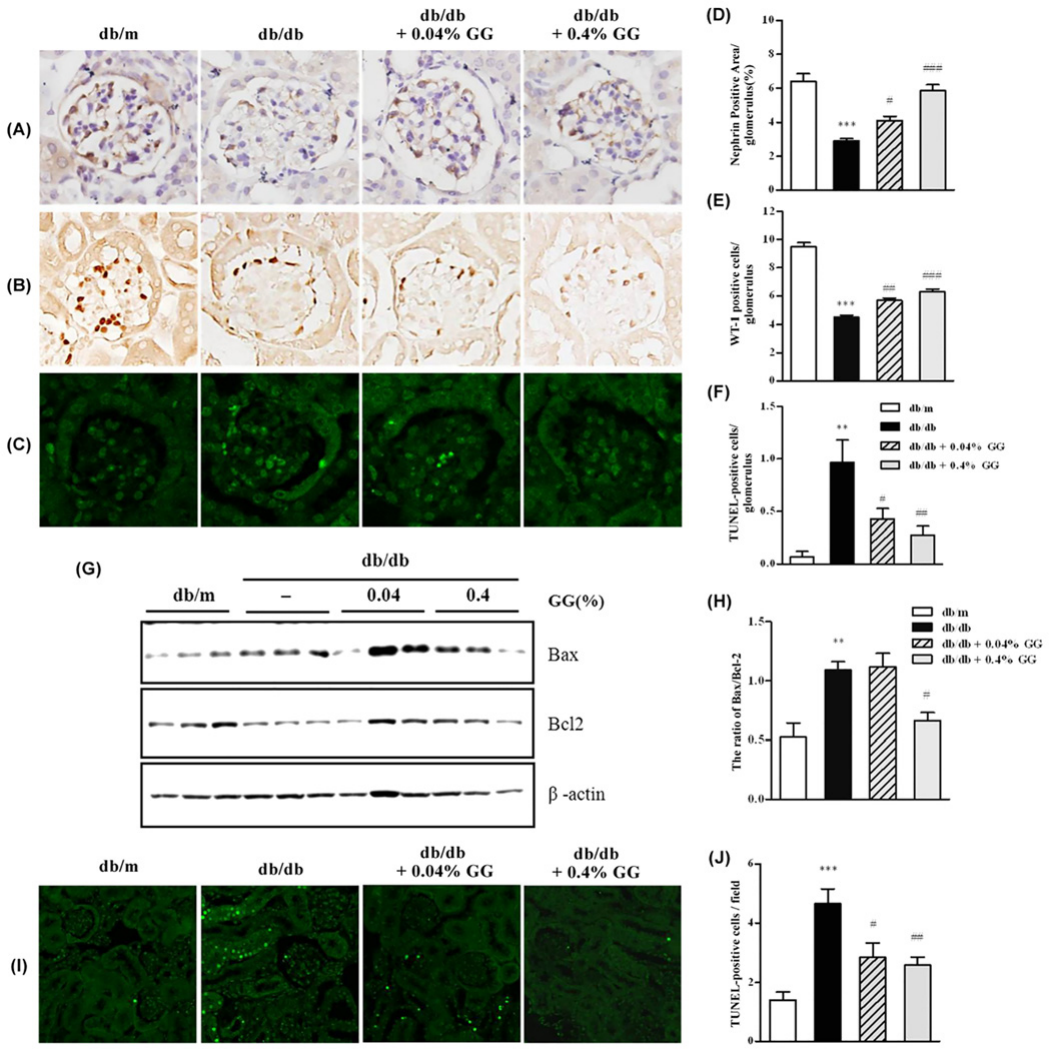
Immunohistochemical staining showing the effect of gemigliptin on injury to podocytes and apoptosis. Representative images of nephrin and WT-1 (A and C) and quantitative analyses of both nephrin and WT-1 levels (B and D) in db/m, db/db, 0.04% (w/w) gemigliptin-treated db/db, and 0.4% (w/w) gemigliptin-treated db/db mice. TUNEL staining detecting apoptotic injury to the glomeruli (E). Quantitative analysis of the numbers of TUNEL-positive cells (F) in the four experimental groups. Analysis of the levels of the pro-apoptotic Bax and anti-apoptotic Bcl2 proteins via Western blotting (G). TUNEL staining detecting injury to the kidney (H). Quantitative analyses of the numbers of TUNEL-positive cells and the Bax/Bcl2 ratios, derived using NIH ImageJ software, are shown for the four experimental groups (I and J). The results are expressed as means ± SEMs. **p < 0.01, ***p < 0.001 vs. db/m; #p < 0.05, ##p < 0.01, ###p < 0.001 vs. db/db. Original magnification ×400.

To explore how gemigliptin reduced albuminuria and mesangial cell expansion when administered at a non-glucoregulatory dose (0.04%), we evaluated the extent of podocyte injury in db/db mice. The diabetes-induced loss of glomerular nephrin was recovered in gemigliptin-treated db/db mice compared with controls (Fig 6A and 6D). The numbers of WT-1 positive cells also increased in both gemigliptin groups (Fig 6B and 6E). The numbers of glomerular apoptotic cells increased in diabetic db/db mice, but these numbers were attenuated by gemigliptin treatment (Fig 6C and 6F). These findings indicate that even the non-glucoregulatory dose of gemigliptin attenuated albuminuria by preventing podocyte injury. We also explored the effect of gemigliptin on apoptotic injury to the kidney tubules of db/db mice. The Bax/Bcl2 ratio in the diabetic kidney was decreased by only 0.4% gemigliptin (Fig 6G and 6H). Apoptotic cell numbers in the tubular region determined by TUNEL staining increased in the db/db kidney, but the number of apoptotic cells was decreased by gemigliptin in a dose dependent manner (Fig 6I and 6J). These findings show that the glucoregulatory dose of 0.4% gemigliptin attenuated diabetic tubular damage by preventing apoptotic injury.

### Only 0.4% Gemigliptin Attenuates NADPH Oxidase Levels and Akt and Foxo3a Pathway in the Diabetic Kidney

**Fig 7:**
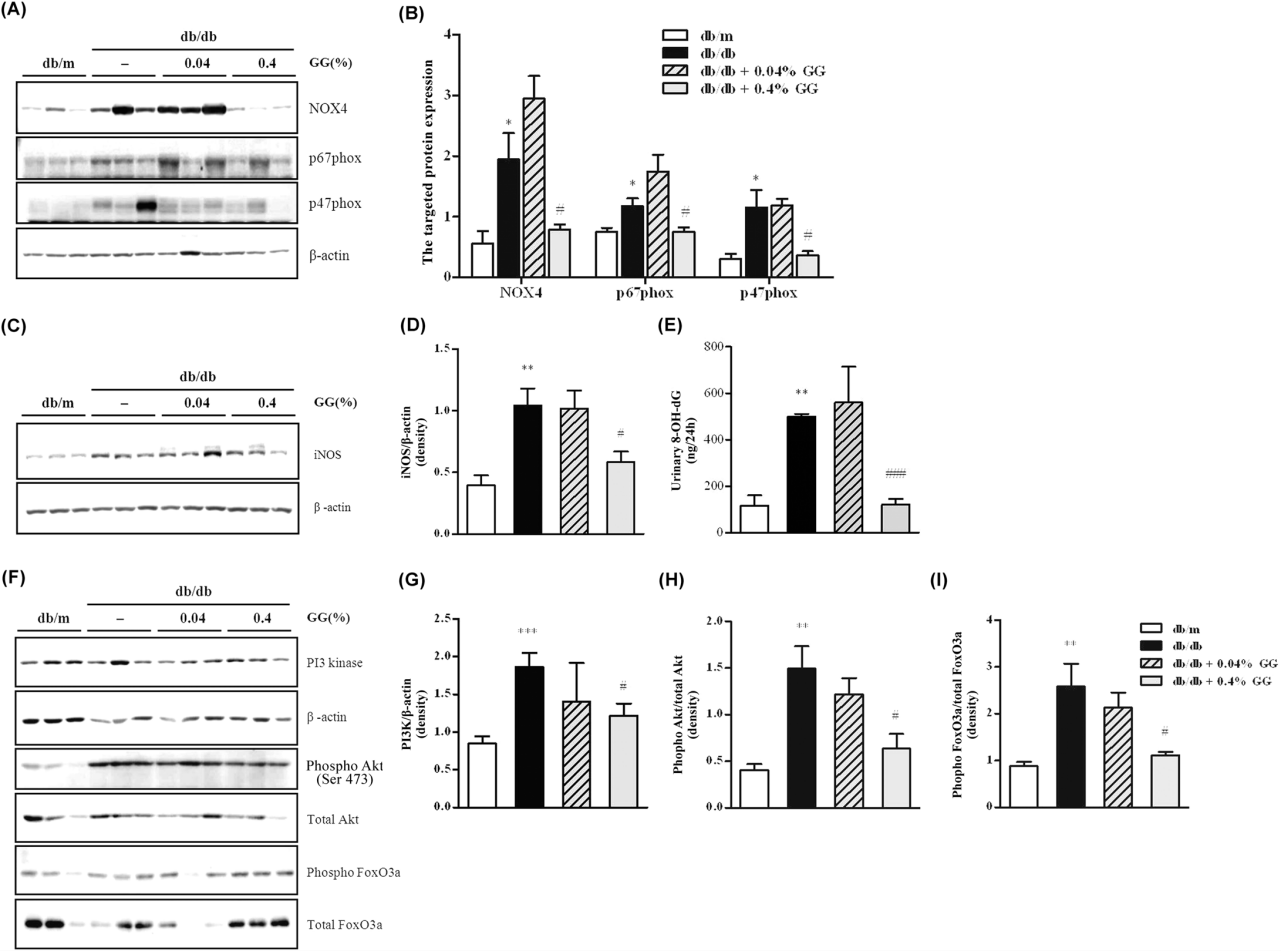
The effects of gemigliptin on NADPH oxidase and Akt and FoxO3a pathways in the kidneys of db/db mice. Representative Western blot analyses of NADPH oxidase (NOX4, p67-phox, and p47-phox) levels and the estimates of ROS-induced injury (including iNO levels) are shown (A and B). NADPH oxidases and ROS-associated enzymes were quantitated using NIH ImageJ software (C and D). Quantitative assessments of urinary 24-h 8-OH-dG levels in each group are shown (E). Representative Western blot showing PI3K, total Akt, phosphor-Akt, total FoxO3a, phosphor-FoxO3a, and β-actin bands (F). The densitometric graphs displaying PI3K/β-actin, pAkt/tAkt, and pFoxO3a/tFoxO3a protein expression ratios derived using NIH ImageJ software (G-I). β-actin served as the internal control. All results are expressed as means ± SEMs. *p < 0.05, **p < 0.01 vs. db/m; #p < 0.05, ###p < 0.001 vs. db/db.

We explored the effect of gemigliptin on the extent of diabetic ROS injury to the kidney. NOX4, p47-phox, and p67-phox of the NAD(P)H oxidase complex were all upregulated in the db/db kidney (Fig 7A and 7B). Such rises were inhibited by 0.4%, but not 0.04% gemigliptin. The levels of iNOs and urinary 8-OHdG were increased in db/db mice and were attenuated only by 0.4% gemigliptin (Fig 7C–7E). Thus, only the glucoregulatory dose of 0.4% gemigliptin attenuated diabetic nephropathy, by decreasing the extent of ROS injury. We explored the effects of gemigliptin on the levels of diabetes-related transcriptional factors in the kidney. The levels of PI3 kinase, phosphorylated Akt, and phosphorylated FoxO3a were elevated in the diabetic kidney (Fig 7F–7I). Gemigliptin attenuated the expression of all three proteins, but this was significant only at the 0.4% concentration.

## Discussion

Our major findings were that gemigliptin, a new DPP-4 inhibitor, had non-glucoregulatory tissue-protective effects on both diabetic nephropathy and cardiomyopathy. However, we also unexpectedly found an ambivalent organ-specific effect in terms of organ damage in the db/db mice model. Gemigliptin protected podocytes in a dose-dependent manner, and the higher dose used exhibited additional anti-apoptotic and anti-oxidant effects in the diabetic kidney. However, gemigliptin exhibited an ambivalent dose-dependent effect on diabetic cardiomyopathy. Low-dose gemigliptin significantly attenuated LV dilatation, myocyte hypertrophy, and cardiac fibrosis, while the higher dose did not show these beneficial effects.

Our present study is relevant to the outcomes of clinical trials reported in 2013. The SAVOR-TIMI 53 trial was associated with a 27% increase in hospitalization (for heart failure) among diabetic patients who received saxagliptin compared with placebo [2]. The heart failure outcomes were not mentioned at all in the report of the EXAMINE trial by White et al., although 28% of patients had congestive heart failure at baseline [3]. Other ongoing trials need to address these heart failure issues. In addition to their anti-glycemic effects, experimental studies have shown that DPP-4 inhibitors exert beneficial cardioprotective effects [5]. It is generally assumed that GLP-1 activation by a DPP-4 inhibitor triggers pro-survival signaling cascades, including the phosphatidylinositol 3-kinase (PI3K)-Akt and cAMP-protein kinase A (PKA) pathways, as responses to acute ischemic reperfusion (IR) injury [6]. Previously, we reported that the GLP-1 inhibitor exenatide potently protected against IR-induced endothelial dysfunction by triggering the opening of human K_ATP_ channels, and reduced myocardial infarct size. The drug exhibited reasonable safety and tolerability in patients with acute myocardial infarction [7,8].

Diabetic cardiomyopathy is associated with cardiac apoptosis, hypertrophy, and fibrosis [9,10].

Our data showed that gemigliptin ameliorated diabetes-induced cardiac apoptosis, which was mediated by GSK3β and NAD(P)H oxidase. However, anti-apoptotic effect of gemigliptin was dose dependent and even abolished in a higher dose. Although Akt-GSK3β plays an important role in insulin signaling pathway and the prevention of cardiac apoptosis, Akt phosphorylation was diminished in diabetic heart and further decreased by both doses of gemigliptin (Fig 4B). However, phosphorylation of GSK3β serine 9 in diabetic heart was increased after treatment with 0.04% gemigliptin. This could be partially explained by the finding that 0.04% gemigliptin treatment activated ERK1/2, another upstream kinase of GSK3β, and thus stimulated GSK3β phosphorylation, even though intracardiac activation of Akt pathway was further decreased by gemigliptin administration. Previous studies showed that inactivation of GSK3β activity by GSK3β-S9A protected cardiomyocytes against oxidant-induced apoptosis [11] and opening of the heart mitochondrial permeability transition pores [12,13]. We just postulate that 0.04% gemigliptin modulates GSK3β levels via ERK1/2 activation. However, effect of DPP4 inhibitor on myocardiac activation of GSK3β pathway and its relevance to the complication of diabetic heart and other target organs should be clarified.

Furthermore, diabetes-associated increases in the expression levels of NAD(P)H oxidase components, including NOX2, p47-phox, and p22-phox, were effectively suppressed by 0.04%, although some component (p47phox) was not affected by 0.4% gemigliptin. These data suggest that 0.04% gemigliptin abolished diabetes-induced cardiac apoptosis, which was mediated by NAD(P)H oxidase. Our data suggests DPP4 inhibitor attenuates myocardiac apoptosis of diabetic heart by the mechanisms of increase of GSK3β phosphorylation and suppression of NAD(P)H oxidase, but this anti-apoptotic effect might be dose dependent and abolished in an extremely higher dose.

Interestingly, in contrary to heart data, gemigliptin showed the reno-protective effect on diabetic kidney injury in a dose dependent manner in our study. GLP-1-independent renoprotective effects have also been reported in streptozotocin-induced and genetic diabetic rodent models [14,15,16]. In previous studies, such effects were suggested to be principally anti-apoptotic and anti-oxidant in nature. In the present study, both doses of gemigliptin evaluated prevented development of albuminuria. To explore how the non-glucoregulatory dose of gemigliptin reduced albuminuria, we evaluated the extent of podocyte injury in db/db mice. We found that gemigliptin preserved nephrin- and podocin-expressing podocytes in a dose-dependent manner. These results indicate that even a non-glucoregulatory dose of gemigliptin could attenuate albuminuria by preventing podocyte injury. We also explored the effects of gemigliptin on apoptosis and oxidative stress and on PI3K/Akt and Foxo3a pathway activities in kidney cortex. Interestingly, the non-glucoregulatory dose exerted minimal effects on these pathways, but high-dose gemigliptin significantly improved renal apoptosis, oxidative stress, and PI3K/Akt-Foxo3a activation. It is strongly suggests that the non-glucoregulatory effect of the DPP-4 inhibitor is associated with podocyte protection, whereas the glucoregulatory dose attenuates diabetic nephropathy by decreasing both apoptotic and ROS-mediated injury and by triggering transcriptional activation affording further glycemic control.

We could not conclude the exact mechanism of organ and dose-specific differences of the effect of DPP4 inhibitor in this study. Organ- and disease-specific variations in DPP-4 expression may also modulate the effects of different DPP-4 inhibitor doses. DPP-4 expression is much higher in kidney tubules than the myocardium [17]. DPP-4 is abundant in the brush borders of kidney proximal tubule cells and is activated further in animal models of diabetes [18,19]. Recently, DPP-4 inhibition has been suggested to be involved in inactivation of TGF-β1 and downstream fibrotic pathways in human proximal tubule cell lines stimulated with high levels of glucose [20]. And shown in our data, activation of critical molecular pathways under diabetic complication is quite different to the target organ. Activation of Akt pathway was suppressed in diabetic heart, but significantly increased in diabetic kidney, although gemigliptin effectively suppressed the phosphorylation of Akt in both target organs of heart and kidney (Figs 3D and 6H), Finally, this animal experiment has some limitations in terms of translation to the clinic. Dosage of gemigliptin used in this study was actually dozen times of clinical dose. On the other hand, we could not investigate whether gemigliptin affect stat3/Pim1 signaling pathway and produces sex difference-related benefits in the experimental conditions. Furthermore, it is unclear whether gemigliptin ameliorates bone-marrow-derived endothelial progenitor cell mobilization and homing into the cardiac vasculature to contribute to the cardiorenal protection. Further study will be needed to investigate these issues. However, this is the first report to show that a DPP-4 inhibitor exerts a dose-dependent, organ-specific effect on the macro- and microvascular complications in type 2 diabetes model.
